# Paranoia, experiential avoidance, and narcissism as predictors of outcome in residential treatment of borderline personality disorder

**DOI:** 10.3389/fpsyt.2026.1728221

**Published:** 2026-03-12

**Authors:** D. Bradford Reich, Lois Choi-Kain, Karen Jacob, Brandon Unruh, Boyu Ren

**Affiliations:** Department of Psychiatry, Harvard Medical School, Boston, MA, United States

**Keywords:** bipolar disorder, borderline personality disorder, experiential avoidance (EA), narcissism, paranoia, residential treatment

## Abstract

**Introduction:**

Previous research has suggested that residential treatment may be effective in reducing symptoms in patients with symptoms of borderline personality disorder (BPD). This study examined whether paranoia, experiential avoidance, and narcissistic personality features predicted response to residential treatment in a cohort of female patients with symptoms of borderline personality disorder.

**Method:**

Participants were 87 women with borderline personality disorder symptoms who participated in multimodal residential treatment incorporating elements of Dialectical Behavioral Therapy, Mentalization Based Therapy, Transference Focused Psychotherapy, and General Psychiatric Management. All participants met definite or probable criteria for borderline personality disorder as assessed by the International Personality Disorder Examination. Participants were evaluated at baseline and at one-month intervals using the Zanarini Rating Scale for Borderline Personality Disorder, Self-Report Version (Zan-BPD, SR), the Paranoid Thoughts Scale (PTS), the Multidimensional Experiential Avoidance Questionnaire (MEAQ), and the Narcissistic Personality Inventory (NPI). The primary outcome measure was change in mean ZAN-BPD scores.

**Results:**

Mean scores on the ZAN-BPD (11 to 7, p<0.001), the PTS (2.4 to 2, p<0.001), the Distress Tolerance Subscale of the MEAQ (3.4 to 2.8, p<0.001), and NPI (1.4 to 1.3, p=0.04) all decreased over time. Higher baseline mean scores on the Social Reference subscale of the PTS and the Distress Aversion Subscale of the MEAQ were associated with larger and faster declines in mean ZAN-BPD scores. Baseline scores on the Behavioral Avoidance subscale of the MEAQ were associated with slower declines in mean ZAN-BPD scores. Longitudinal declines in the Distress Aversion subscale of the MEAQ and the Social Reference subscale of the PTS were significantly associated with reductions in mean ZAN-BPD scores over time. NPI scores were not significantly associated with changes in ZAN-BPD scores.

**Conclusion:**

Multimodal residential treatment may be an effective treatment for female patients with borderline personality disorder. Paranoia and experiential avoidance may not be negative prognostic factors in the treatment of such patients and should be considered significant residential treatment targets. Narcissistic personality features may change more slowly and appear to be less influential in residential treatment for borderline personality disorder.

## Introduction

Borderline personality disorder has a prevalence rate of 0.7-2.7%. It is common in outpatient and inpatient clinical settings with prevalence rates of 11-12% and 22%, respectively (1). Although many patients with borderline personality achieve remission of symptoms and functional recovery from the disorder, a substantial number of borderline patients do not achieve stable recovery (2). This suggests that more intensive treatments, such as treatment in a residential setting, might be beneficial to patients with more severe cases of BPD.

Multiple studies have found that intensive residential treatment may reduce symptoms of personality disorders and BPD, in particular (3–9). These studies have found significant improvement in borderline symptoms (9), anxiety (4, 8), depression (4, 8), reflective functioning (6), self-injury (4), overall level of psychiatric symptoms (3, 4, 6, 7), and overall functioning (4). It should be noted that several of these studies found that residential treatment alone was no more or marginally more effective than outpatient treatment (3, 10) or that it was necessary to pair residential treatment with outpatient follow-up to achieve significantly improved outcomes over outpatient treatment alone (7). The one study examining predictors of response to residential treatment in personality disordered patients found that a history of self-injury, avoidant personality disorder, and older age were associated with worse residential treatment outcomes while length of treatment and lower severity of symptom scores were associated with better outcomes (11).

Paranoia, experiential avoidance, and pathological narcissism, have all been associated with BPD (12–14). Nondelusional paranoia is a DSM 5 criterion for BPD and appears in both clinical and subclinical forms of BPD (12, 15). Paranoid symptoms, such as distrust of others’ intentions and ideas of reference, occur more commonly in BPD, as well as schizotypal personality disorder and paranoid personality disorder, than in other personality disorders (12). Paranoia in BPD appears reactive and thus not stable, possibly because borderline individuals have fluctuating perceptions of others (16, 17). Research has found that undue suspiciousness, ideas of reference and other forms of paranoia decrease but continue to be reported by a substantial percentage of borderline individuals over time (18). Reducing paranoia in borderline patients may be important because paranoia may significantly interfere with the ability of borderline patients to form trusting relationships, both in and outside of treatment.

Experiential avoidance is the tendency to avoid remaining in contact with private experiences (bodily sensations, emotions, thoughts, memories, and behavioral dispositions) and acting to modify the form, the frequency, or the context of these experiences. It occurs even when it may cause behavioral harm (19). Although not a DSM 5 criterion for BPD, experiential avoidance is frequently associated with it. This association appears to be independent of difficulties with emotion regulation and distress tolerance (20). Borderline individuals report avoiding situations, activities, places, and people associated with anxiety more than individuals with other personality disorders. In addition, they report that anxiety is more likely to interfere with vocational, social, and overall functioning than individuals with other personality disorders (13). In borderline patients receiving dialectical behavioral therapy, reductions in experiential avoidance have been associated with reductions in depression, both concurrently and prospectively (21). Thus, higher levels of experiential avoidance might negatively influence the responsiveness of patients with BPD to psychosocial treatment.

Narcissistic personality disorder (NPD) is frequently comorbid with BPD. NPD may co-occur in approximately 40% of individuals with BPD (22). BPD tends to be more closely associated with vulnerable rather than grandiose narcissism (14). Research on narcissistic personality disorder has suggested that it may be difficult to treat for multiple reasons. Two thirds of patients in treatment for NPD leave treatment prematurely (23). Furthermore, the dismissive attachment style associated with NPD may undermine therapeutic alliances, weaken commitment to treatment, and predispose narcissistic patients to avoid bringing relevant issues into treatment. Finally, the perfectionism, vulnerability to shame, and proclivity to engage in devaluation associated with pathological narcissism may prevent patients with NPD from using treatment effectively (24). These findings suggest that NPD features may complicate treatment of borderline personality disorder.

This study investigated the possible impact of paranoia, experiential avoidance, and narcissism on outcomes in residential treatment for patients with borderline personality disorder. We chose these features not only because they are associated with BPD, but because both research and clinical experience suggest that they may interfere with borderline patients’ paths to recovery. We hypothesized that:

1. Higher baseline levels of paranoia, experiential avoidance, and narcissism would correlate with smaller responses of BPD symptoms to treatment
2. Higher baseline levels of paranoia, experiential avoidance, and narcissism would correlate with slower responses of BPD symptoms to treatment
3. Longitudinal improvements in paranoia, experiential avoidance, and narcissism would correlate with longitudinal responses of BPD symptoms to treatment

## Methods

### Participants

Participants were drawn from 121 patients consecutively admitted to a self-pay residential treatment program for women with personality disorders. Patients were included in the study if they:1) met DSM 5 criteria or probable criteria for borderline personality disorder as assessed by the International Personality Disorders Examination and 2) remained in the program for at least one month (long enough to complete at least one post-baseline battery of assessments). Eighty-one participants met definite or probable DSM 5 criteria for BPD. More specifically, the sample was comprised of 71 participants who met DSM-5 criteria for BPD and 10 participants who met probable DSM 5 criteria for BPD (meeting 4 BPD criteria). The mean age of the sample was 27. All participants had previously been in outpatient treatment. Fifty-five percent of the sample had been psychiatrically hospitalized at least once and 34% had been psychiatrically hospitalized at least twice. Sixty-five percent had been in at least one previous residential treatment and 39% had been in two or more previous residential treatments. Sixteen percent of the sample had attended a partial hospitalization program; 13% had attended an intensive outpatient program. As detailed in **Tables 1**, **2**, most participants met definite or probable criteria for multiple DSM 5 personality disorders.

### Procedure

The study assessed participants diagnostically using the MINI International Neuropsychiatric Interview (MINI) (25) and the International Personality Disorder Examination. The primary outcome measure for the study were scores on the Zanarini Rating Scale for Borderline Personality Disorder (ZAN-BPD). This was administered at baseline and then monthly while patients were in the program. The study obtained assessments of paranoia, experiential avoidance, and narcissism at baseline and then at one-month intervals. Paranoia was assessed using the Paranoid Thoughts Scale (PTS). Experiential avoidance was assessed using the Multidimensional Experiential Avoidance Questionnaire (MEAQ). Narcissism was assessed using the Narcissistic Personality Inventory (NPI). All study data were collected as part of standard clinical assessment in the residential treatment program described below. This study and retrospective analysis of study data were classified as exempt and approved by the Mass General Brigham Institutional Review Board as part of routine clinical quality assurance. The study was not pre-registered.

### Treatment

Treatment consisted of 3–6 months of a milieu based residential treatment program designed for women with severe personality disorders who had not responded to outpatient treatment. The program involved daily group therapy sessions, twice weekly individual therapy, weekly family therapy, and weekly psychopharmacologic monitoring. The program combined principles of various evidence-based treatments for borderline personality disorder, including Dialectical Behavioral Therapy (DBT), Cognitive Behavioral Therapy (CBT), Mentalization-Based Treatment (MBT), and Good Psychiatric Management of BPD (GPM). It presented patients with key elements of each approach to help them learn more effective ways of managing their thoughts, emotions, behaviors, and interpersonal relationships while placing an overall treatment emphasis on the importance of building a life outside of treatment through improved functioning in normative social and vocational roles. Central to the program was principle that patients’ psychopathology would improve only if they were confronted with feedback about it both by program clinicians and other patients in the program. In this way the program tried to encourage patients not to engage in experiential avoidance.

DBT was implemented in the program with adherence to the model developed by Marsha Linehan and her colleagues. Patients learned DBT skills through four weekly skills groups dedicated to each of the four skills modules: Distress Tolerance, Emotion Regulation, Interpersonal Skills, and Mindfulness. Individual psychotherapy with a primary therapist was highly DBT-oriented, implementing principles of behavioral shaping. At the primary therapist’s discretion, individual psychotherapy might also incorporate elements of MBT and TFP. All clinical staff participated in a weekly consultation team led by a nationally renowned Behavioral Tech DBT trainer in accordance with Linehan’s original model.

In the program, patients attended two weekly MBT groups to help them develop and strengthen their ability to understand multiple viewpoints and move toward an understanding that different people may hold different perspectives and ways of thinking about situations. In addition, patients attended weekly CBT and Self-Assessment groups where they learned the interactive relationship between thoughts, feeling and behaviors in order to develop a more structured self-awareness. The program expected patients to apply their conceptual understanding of this relationship to situations occurring in the therapeutic milieu. The program also expected patients to use a number of CBT tools, such as mood monitoring and behavioral scheduling, to increase their own awareness of this interactive relationship and how it pertained to their own lives.

### Clinical measures

#### Paranoid thoughts scale

The Paranoid Thoughts Scale is a 32-item self-report measure assessing a spectrum of paranoid symptoms. It measures paranoia on dimensions of preoccupation, conviction, and distress. It contains items measuring both social reference, paranoid thoughts and persecutory beliefs. It asks respondents to rate paranoid experiences over the last month on a 5-point Likert scale. It contains two subscales, one measuring persecutory beliefs and one measuring social reference. It is designed to be used with both clinician and non-clinical populations (26).

#### Narcissistic personality inventory

The Narcissistic Personality Inventory is a 40 item self-report measure. It contains 40 items measuring both adaptive and maladaptive narcissism. Each item asks respondents to endorse one of two opposing statements about their perception of themselves. Dimensions contained in the instrument include vanity, entitlement, authority, self-sufficiency, superiority, exploitativeness and exhibitionism (27).

#### Multidimensional experiential avoidance questionnaire

The Multidimensional Experiential Avoidance Questionnaire (MEAQ) is a 62-item self-report. It contains 6 subscales measuring: 1) Distress Avoidance; 2) Distress Endurance; 3) Behavioral Avoidance; 4) Suppression/Distraction; 5) Repression/Denial; and 6) Procrastination. It has been validated in both clinical and normal populations. Research has shown it to have good internal consistency, convergent validity, and discriminant validity (28).

#### Zanarini rating scale for borderline personality disorder

The Zanarini Rating Scale for Borderline Personality Self Report Version (Zan-BPD SR) measures each of the nine DSM-5 criteria for borderline personality disorder within a one-week time frame. Each of the nine criteria is rated on a five-point anchored rating scale from 0-4. The instrument has good internal consistency and sensitivity to change (29).

#### International personality disorders examination

The International Personality Disorders Examination (IPDE). The IPDE is a semi-structured interview designed to provide personality disorder diagnoses compatible with the DSM-IIIR and ICD-10. It contains 157 items. Each item is scored on a 3-point scale ranging from 0 – Absent or within normal range; 1- present to an accentuated degree; 2- pathological, meets criterion. It provides both a categorical and a dimensional score for each disorder. Scoring of the instrument requires that a behavior or trait be present for 5 years to be considered present. In addition, it requires that to meet criteria for a disorder, at least one criterion be present before age 25. To meet full criteria for a personality disorder, the instrument requires that 5 criteria for that personality disorder to be met; to receive a “probable” diagnosis of a personality disorder, the instrument requires that 3 criteria for that personality disorder be met (30).

### Statistical analyses

To examine how baseline profiles of paranoia, experiential avoidance, and narcissistic personality relate to overall changes in BPD symptoms after the residential program, two linear models were considered. In the first model, the change in ZAN-BPD score from baseline to end of treatment was used as the outcome. Covariates included baseline total scores of the NPI, the PTS, and three MEAQ subscales: distress aversion, distress endurance, and behavioral avoidance. The second model accounted for variation in patients’ length of stay by using the *rate* of change in ZAN-BPD score as the outcome. This rate was estimated via a linear mixed model (LMM), with ZAN-BPD scores as the outcome and time since baseline as the sole covariate. Subject-specific random intercepts and slopes were included in the model to capture variations of temporal trends of ZAN-BPD across subjects. The estimated rate of change was defined as the individual-level regression coefficient for time. For both models, a sensitivity analysis with PTS replaced by two of its subscales, Persecution and Social Reference, was performed.

Beyond baseline predictors of symptom change, we also examined longitudinal associations between BPD symptoms and the three psychological domains. An additional LMM was fit using ZAN-BPD scores as the outcome, with total NPI, two subscales of PTS (Persecution and Social Reference), and the three MEAQ subscales as time-varying covariates. Time since baseline was included to account for the natural progression of symptoms during residential treatment, and subject-specific random intercepts and slopes were included to capture intra-subject correlations.

Finally, to identify patients who experienced a clinically meaningful improvement in BPD symptoms, we applied the Reliable Change Index (RCI) analysis to ZAN-BPD scores (31). This method accounts for measurement error and determines whether an individual’s change in score exceeds what would be expected by chance. Patients were classified as having a reliable change (versus no reliable change) based on this index. A logistic regression was then performed to examine whether baseline NPI, PTS and MEAQ scores were associated with the status of reliable change. All analyses were conducted in R (version 4.4.2) (32). The R package lme4 was used to fit linear mixed models. R packages dplyr, ggplot2, and tidyr were used for data manipulation and visualization.

## Results

As detailed in **Table 1**, approximately 90% of the sample met full criteria for borderline personality disorder and 10% of the sample met probable criteria for borderline personality disorder. The sample contained significant comorbidity. Approximately one-third of participants met criteria for obsessive compulsive personality disorder or avoidant personality disorder; and approximately one-quarter of the sample met criteria for narcissistic personality disorder. Twenty participants met criteria for 2 personality disorders, 18 met criteria for 3 personality disorders, 10 met criteria for 4 personality disorders, and 5 met criteria for 5 or more personality disorders.

**Table 1:** Personality disorders in study group.

Full criteria for DSM 5 personality disorder	N(%)
Borderline	71 (88)
Avoidant	30 (37)
Obsessive-Compulsive	28 (35)
Narcissistic	20 (25)
Dependent	10 (12)
Paranoid	10 (12)
Histrionic	7(9)
Antisocial	5(6)
Schizotypal	1(1)
Schizoid	1(1)
Criteria for probable DSM 5 personality disorder	
Borderline	10(11)
Narcissistic	8(10)
Avoidant	12(15)
Obsessive Compulsive	12(15)
Antisocial	4(5)
Dependent	13(16)

As might be expected, participants in the study met criteria for multiple non-personality disorder psychiatric disorders. As shown in **Table 2**, the most common of these was major depressive disorder, occurring in over 50% of participants. Consistent with previous studies of the overlap between bipolar disorder and borderline personality disorder, almost 20% of participants met criteria for bipolar 1 or bipolar 2 disorder. Anxiety disorders were common among participants and occurred in between 15% and 40% of the sample. Substance or alcohol use disorders were also common: over one quarter of participants met criteria for either a substance abuse or alcohol abuse disorder. Approximately 20% of the sample met criteria for an eating disorder, predominantly bulimia or binge eating disorder. 65% of the sample endorsed having at least two types of potentially self-damaging behaviors on the IPDE item measuring general impulsivity, suggesting that the sample had both internalizing and externalizing symptoms.

**Table 2:** Non-personality disorder diagnoses in study group.

Diagnosis	N(%)
MDD	50(62)
Bipolar I	12(15)
Bipolar II Disorder	2(2)
ETOH Use Disorder	22(27)
Substance Use Disorder	24(30)
Anorexia Nervosa	1(1)
Bulimia Nervosa	9(11)
Binge Eating Disorder	8(10)
Social Anxiety Disorder	23(28)
Generalized Anxiety Disorder	32(40)
OCD	15(19)
Panic Disorder	19(23)
PTSD	18(22)
Psychosis NOS	2(2)
Screened only for Personality Disorders	10(12)

**Figure 1:**
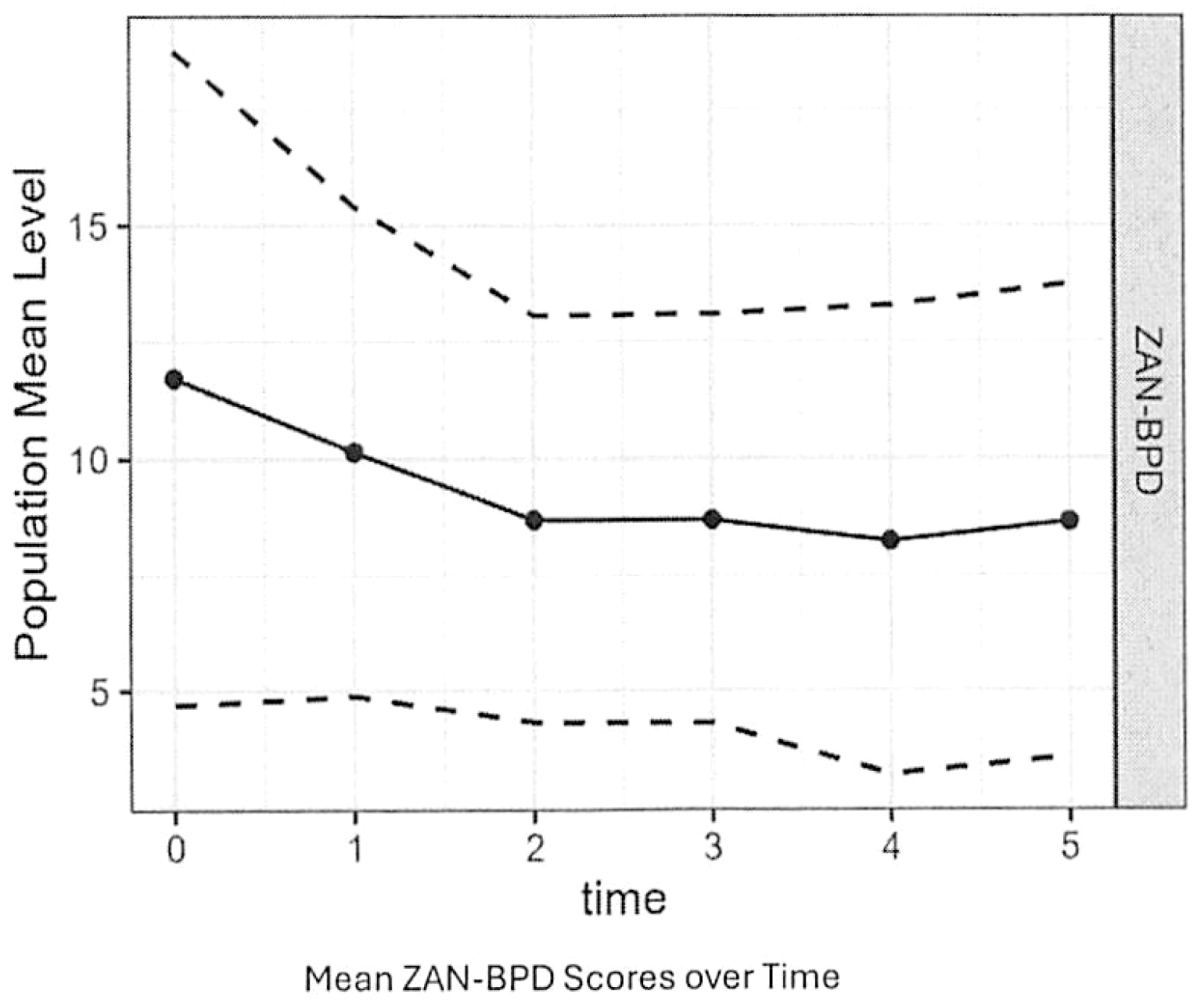
Mean ZAN-BPD scores over time.

ZAN-BPD, NPI, MEAQ, and PTS scores all declined significantly over time. But the rate of decrease in NPI scores were significantly less than for scores on the ZAN-BPD and the other two outcome variables. **Figure 1** plots mean ZAN-BPD scores over time. The mean ZAN-BPD score decreased from 11 at baseline to 7 by follow-up period 5 (rate of change -0.86, SE 0.19, p<0.001). Most of the decrease in ZAN-BPD scores occurred between baseline and the end of the second month of treatment. The mean PTS score decreased from 2.4 at baseline to 2 by follow-up period 5 (rate of change -0.18, SE = 0.03, p-value<0.001). The mean MEAQ score declined from 4.5 at baseline to 4 by follow-up period 5 (rate of change -0.18, SE = 0.03, p-value<0.001). The mean total NPI score decreased from 1.4at baseline to 1.3 by follow-up period 5 (rate of change -0.06, SE = 0.03, p-value=0.04).

**Figure 2:**
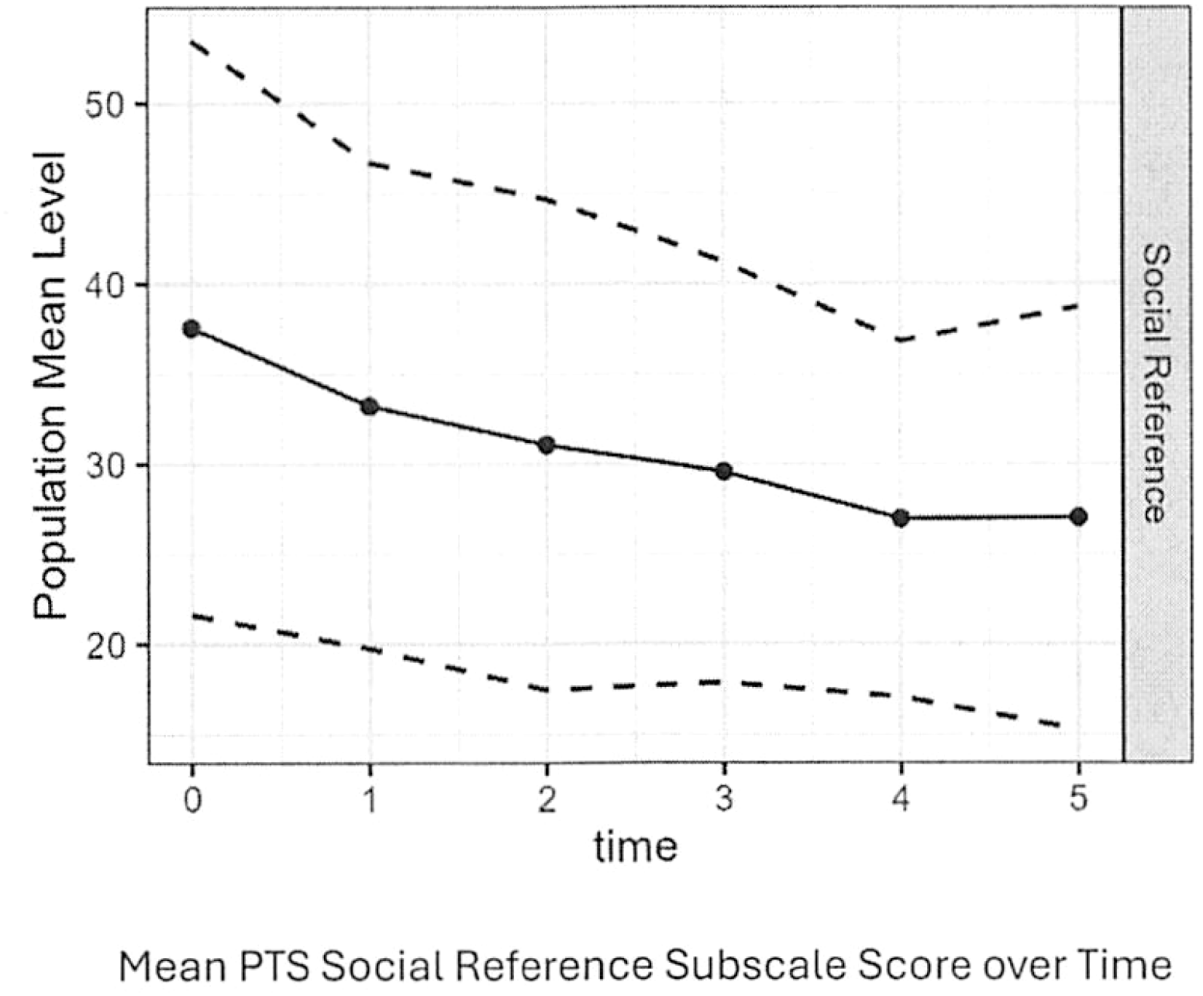
Mean PTS social reference subscale score over time.

**Figure 3:**
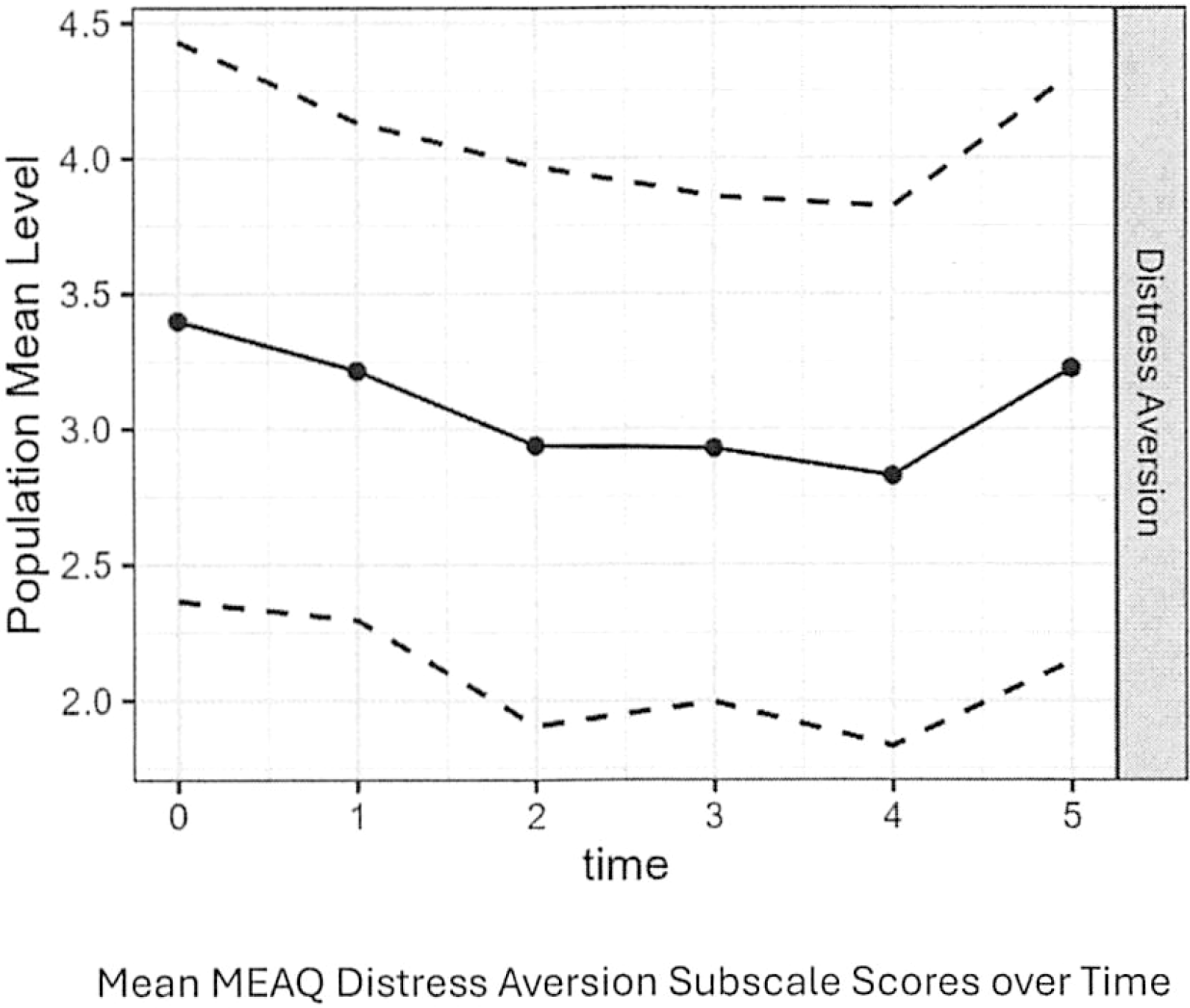
Mean MEAQ distress aversion subscale scores over time.

Mean PTS Social Reference subscale scores and mean MEAQ Distress Aversion Scores decreased significantly over time, but the former decreased more than the latter. As shown in **Figure 2**, mean Social Reference Subscale scores of the PTS decreased from 37.5 at baseline to 27.1 by follow-up period 5 (rate of change -2.89, SE = 0.41, p-value<0.001). As shown in **Figure 3**, mean MEAQ Distress Aversion subscale scores declined from 3.4 at baseline to 2.8 by follow-up period 4 (rate of change -0.16, SE = 0.03, p-value<0.001) before increasing during the last follow-up period.

**Table 3** displays multivariate baseline predictors of reductions in ZAN-BPD scores. The model includes baseline total PTS, baseline NPI scores, baseline MEAQ scores, and baseline scores for 3 subscales of the MEAQ associated with distress. In this model only baseline PTS scores and baseline scores on the Distress Aversion subscale of the MEAQ were significantly associated with decreases in the ZAN-BPD. A separate analysis using baseline scores of the Persecution and Social Reference subscales of the PTS did not find either of these scores significantly associated with changes in the ZAN-BPD.

**Table 3:** Baseline predictors of change of ZAN-BPD scores from baseline.

Predictor	Estimate std	SE	Confidence interval	P-Value
NPI Baseline	−0.92	0.75	−2.42 to −0.59	0.23
PTS Baseline	−2.35	0.83	−4.02 to −0.69	0.006
Distress Aversion	−3.15	1.07	−5.72 to −0.58	0.012
Distress Endurance	−0.27	0.82	−1.91 to −1.37	0.74
Behavioral Avoidance	1.89	1.14	−0.40 to −4.17	0.10

**Table 4** presents baseline multivariate predictors of faster declines in ZAN-BPD scores. Higher baseline scores on the PTS and Distress Aversion subscale if the MEAQ were significantly associated with faster decreases in mean ZAN-BPD scores. In addition, higher baseline scores on the Behavioral Avoidance subscale of the MEAQ emerged as a significant predictor of slower declines in mean ZAN-BPD scores.

**Table 4:** Baseline predictors of rate of change of ZAN-BPD scores.

Predictor	Estimate std	SE	Confidence interval	P-Value
NPI Baseline	0.29	0.33	−0.38 to −0.96	0.39
PTS Baseline	−0.88	0.36	−1.59 to −0.17	0.016
Distress Aversion	−1.29	0.46	−2.21 to −0.37	0.007
Distress Endurance	−0.08	0.36	−0.80 to 0.65	0.83
Behavioral Avoidance	1.03	0.49	0.06 to 2.01	0.038

**Table 5** reports the longitudinal associations between ZAN-BPD scores and the six predictors used in the previous two models. As with baseline predictors in **Table 3**, decreases in scores on the Social Reference subscale of the PTS and the Distress Aversion subscale of the MEAQ were the only two variables positively associated with reductions in ZAN-BPD scores.

**Table 5:** Longitudinal association between baseline predictors of change and ZAN-BPD scores.

Predictor	Estimate std	SE	Confidence interval	P-Value
NPI	0.20	0.37	−0.53 to 0.93	.60
PTS Persecution	0.02	0.03	−0.04 to 0.08	0.48
PTS Social Reference	0.12	0.03	0.06 to 0.18	<0.001
MEAQ Distress Aversion	1.10	0.43	0.25 to 1.95	0.012
MEAQ Distress Endurance	−0.54	0.40	−1.32 to 0.25	0.18
MEAQ Behavioral Avoidance	0.22	0.45	−0.66 to 1.10	0.62

The results based on the binary outcome of reliable change in BPD symptoms, however, revealed that none of the total scores on the PTS, NPI, and MEAQ were significantly associated with reliable changes in ZAN-BPD scores.

## Discussion

This study found that over time, residential treatment was associated with improvements in scores measuring borderline symptoms, narcissistic symptoms, paranoia, and experiential avoidance. Patients who reported higher levels of paranoia and distress aversion at baseline had larger improvements in reported BPD symptoms. Patients reporting higher baseline levels of paranoia and distress aversion reported faster declines in BPD symptoms, whereas patients reporting higher baseline levels of baseline behavioral avoidance reported slower improvement. Longitudinal decreases in distress aversion and social reference tracked longitudinal decreases in borderline symptoms. Features of narcissism measured by the NPI were not significantly associated with patients’ report of improvements as measured by the ZAN-BPD. Notably, both predictors of declines in borderline symptoms were internalizing symptoms.

Our results suggest that effective residential treatment of borderline personality disorder may either directly or indirectly target symptoms related to paranoia and aspects of experiential avoidance. The strongest predictor associated with improvement in borderline symptoms were scores on the PTS. This predictor appeared to interact with borderline symptoms in multiple ways. Contrary to expectations, higher levels of baseline paranoia were associated with larger and faster declines in borderline symptoms. As expected, declines in reported paranoid social reference paralleled declines in reported borderline symptoms. In one sense, the positive association between baseline paranoia scores and improvements in ZAN-BPD scores is counterintuitive to the extent that paranoia might impair patients’ ability to accept feedback from a therapeutic milieu or to form a therapeutic alliance with treaters. But study results suggest that at least some patients can respond to interventions that either directly or indirectly address paranoia, particularly paranoid social reference, early in residential treatment and that this may lead to both faster and larger reductions in borderline symptoms. Beyond that, study results suggest that targeting paranoid social reference over the course of residential treatment for borderline patients may be associated with more robust outcomes.

Treatment provided to participants in the study may have reduced paranoia in multiple ways. First, forcing patients to socialize in a supportive, predictable therapeutic milieu may in itself have encouraged borderline patients to be more trusting of others. Second, elements of MBT may have reduced certain kinds of paranoia by improving patients’ capacity to mentalize others. Third, DBT may have reduced paranoid social reference by encouraging patients to use the emotion regulation skill of “checking the facts.” Fourth, it is possible that living in a milieu that consistently requires social exposure while being in a dyadic relationship with a therapist allows the therapist to use evidence-based therapeutic techniques to help patients manage social challenges and thereby promotes a therapeutic alliance that rapidly reduces paranoia.

Paranoia is a complex, hierarchical concept: milder forms include social anxiety and fear of negative evaluation by others (fears of social rejection, thoughts that world is an unsafe place); more intermediate forms include distrust of others’ intentions or ideas of reference; and more severe forms center on persecutory delusions (12). The PTS includes items covering all these categories (26). Clinical experience suggests that borderline patients are more vulnerable to milder forms of paranoia, typically involving social reference, and that these might be more amenable to psychotherapeutic interventions. Results of the study do not suggest that, compared to persecutory beliefs. milder forms of paranoia involving social reference predict larger or more rapid responses to residential treatment. They do, however, suggest that reductions in milder forms of paranoia parallel improvements in borderline symptoms in a residential setting.

Experiential avoidance, although less robustly correlated with reductions in self-reported borderline symptoms in our study, may have been an equally important factor in response to treatment. Contrary to expectations, higher baseline scores on the Distress Aversion subscale of the MEAQ correlated with larger and more rapid improvements in ZAN-BPD scores. Consistent with expectations, higher Behavioral Avoidance scores were associated with slower declines in ZAN-BPD scores. Also consistent with expectations, decreases in Distress Aversion paralleled decreases in ZAN-BPD score. It is not surprising that intolerance of distress is a factor in treatment outcomes for borderline patients. This characteristic is most likely a significant factor in maintaining borderline psychopathology (33). As patients are better able to tolerate distress, they are better able to regulate their emotions and be more effective interpersonally. As with paranoid symptoms, results of the study suggest that using behavioral or cognitive interventions targeted toward reducing intolerance of distress may have facilitated improvements in self-reported borderline symptoms. It should be noted that mean distress aversion scores appeared to increase during the last follow-up period. A likely explanation for this is that patients who had experienced significant declines in distress aversion were more likely to have been discharged from the program before completing 5 months of residential treatment and that those patients still in the program had higher levels of distress aversion. By follow-up period 5, only 23% of participants who had filled out baseline MEAQ scores were still in the program.

In the study, reductions in self-reported distress aversion were most significantly associated with improvement in borderline symptoms. Improvements in behavioral avoidance predicted slower improvement and did not occur in association with reductions in borderline symptoms. This suggests that changes in patients’ internal orientation toward distress – i.e. thoughts and feelings related to distress may be an earlier predictor of response to treatment. Furthermore, the absence of any correlation between distress endurance and improvement in borderline symptoms suggests that a more active orientation toward managing internal and external aspects of distress may occur later in treatment and may not be necessary for the initial stages of a treatment response begun in a residential setting.

Although reported narcissistic pathology declined in our study, it did so at a slower rate than scores related to distress aversion and paranoia. Moreover, contrary to our hypotheses, reported narcissistic pathology was not significantly associated with outcomes in reported borderline symptoms. One possible explanation for this is that changes in narcissism are inherently less amenable to change in the 2-6-month window of treatment assessed in our study. A second possible explanation is that, even when narcissistic pathology does change, it has less impact on core borderline symptoms than changes in paranoia and certain aspects of experiential avoidance. A third possibility is that our predictor measure, the NPI, primarily measures grandiose narcissism rather than vulnerable narcissism, thought more closely tied to BPD (14). It is unlikely that the weaker link between narcissistic pathology and borderline symptoms resulted from weaker targeting of narcissism by clinicians in the program where participants received treatment. Program Clinicians were highly attuned to narcissistic pathology and had substantial expertise in treating it. Nevertheless, the relatively weak association between narcissistic features and improvement in borderline symptoms in our study should not be interpreted as evidence that narcissism should not be centrally targeted in the treatment of borderline personality disorder, residential or otherwise. Rather, it should be seen as consistent with clinical experience that narcissistic pathology may take significantly longer to respond to treatment than borderline symptoms. The 2-6-month window of treatment covered by the study may simply not have been long enough to capture more significant improvements in narcissism.

Results of the study are generally consistent with previous research finding that residential treatment was effective in reducing borderline symptoms (3–9). But our results are not necessarily comparable to previous studies for multiple reasons. First, the one previous study that used the ZAN-BPD as an outcome measure included only adolescents assessed after one-month of residential treatment (9). Second, previous studies assessed outcomes between 1 and 42 months after patients had been discharged from residential treatment. Third, four of these studies reported outcomes of 3–6 months of residential treatment followed by 6–18 months of outpatient stepdown (3, 5–7). Third, the residential treatment provided in our study was based on a hybrid model incorporating many elements of DBT, but also components of mentalization based therapy, transference-based psychotherapy, and general psychiatric management.

To our knowledge, this is the first study to examine the correlation between paranoia and symptomatic outcomes in residential treatment of borderline personality disorder. Previous research on the relationship between BPD and paranoia has been in outpatient samples (18, 34). Such research is consistent with our results to the extent that it found that paranoia is not stable (17) and appears to decline along with other borderline symptoms over time. But previous research reported declines in borderline paranoid symptoms over intervals of at least two years and did not associate declines in paranoid symptoms with treatment status.

As with paranoia, this is the first study in our knowledge to assess the course of experiential avoidance in residential treatment of patients with severe personality disorders. Moreover, it is the first study to examine the relationship between such treatment and individual dimensions of experiential avoidance. Our results align with previous research showing that avoidant personality traits are significant predictors of outcome in residential treatment followed by outpatient stepdown (11); but it does not support the theory that avoidance generally interferes with residential treatment. In fact, our results suggest that residential treatment may be effective in reducing avoidance and thereby improving residential treatment outcomes.

This study had multiple limitations. One set of limitations involved the study sample. Although all participants in the study had personality disorders containing at least some borderline features, most participants met criteria for multiple personality disorders, as well as other psychiatric disorders; and approximately 10% of participants did not meet full criteria for BPD. Beyond that, the study sample included only women. Finally, our sample size was not large enough to find significant correlations between individual BPD symptoms and predictors. Nor was the sample large enough to find correlations between the ZAN-BPD and subscales or individual items on the PTS or NPI. Perhaps because of the small sample size, reliable change analysis did not find a significant association between changes in the ZAN-BPD and the three predictor variables. A second set of limitations involved assessment. Logistical constraints made it impossible to obtain follow-up assessments after participants had been discharged from the program. This prevented evaluating whether gains in the program were sustained. In addition, all assessment instruments in the study were self-report. A third set of limitations involved the therapeutic intervention itself. Treatment in the program was based on a hybrid model that included MBT, TFP and other modalities, as well as DBT, making it difficult to identify which therapeutic factors may have contributed most to change in the variables studied. Equally important, it is not clear that results of the study can be applied to outpatient treatment, the modality used for most patients with borderline personality disorder. These limitations suggest that future research might include a larger sample size, drawn from more diverse socioeconomic backgrounds, and treatment that has both residential and outpatient components, as well as more long-term follow-up.

A central limitation of the study was the socioeconomic status of the sample. Most of the women treated in our residential program were from affluent backgrounds, providing them with resources not available to most patients. This might significantly limit the generalizability of our findings to more typical populations of patients with borderline personality disorder features.

In conclusion, results of the study suggest that residential treatment may be an effective modality for at least a limited set of patients with borderline personality disorder. They further suggest that paranoid ideation and experiential avoidance are not negative prognostic factors in the treatment of such patients and should be considered significant residential treatment targets. Narcissistic personality features may change more slowly and appear to be less influential in residential treatment.

## Data Availability

The datasets presented in this article are not readily available because release of any data will need to be approved by Mass General Brigham Institutional Review Board. Requests to access the datasets should be directed to breich@mclean.harvard.edu.
